# Bidirectional Associations Between Depressive Symptoms and Cognitive Functioning in Older Adults: Heterogeneity by Grandparenthood Status

**DOI:** 10.3390/jintelligence14080159

**Published:** 2026-08-01

**Authors:** Javier López, Ana Belén Navarro, Juan Carlos Meléndez, Miguel Angel Millan

**Affiliations:** 1 Department of Psychology, School of Medicine, Universidad San Pablo-CEU, CEU Universities, 28003 Madrid, Spain; 2 Department of Developmental Psychology, Faculty of Psychology, Universidad de Salamanca, 37008 Salamanca, Spain; 3 Department of Developmental Psychology, Faculty of Psychology, Universitat de Valencia, 46010 Valencia, Spain; melendez@uv.es; 4 Department of Humanities, ESIC University, 28223 Pozuelo de Alarcón, Madrid, Spain

**Keywords:** ageing, cognitive ageing, cognitive functioning, depressive symptoms, EURO-D, grandparenthood, longitudinal analysis, older adults

## Abstract

Depressive symptoms and cognitive functioning are intertwined in later life, but it is unclear whether their time-ordered associations differ by grandparenthood status. Using SHARE (2006–2022) data from adults aged ≥50 with consecutive observations, we fit lagged linear mixed-effects models with person-specific random intercepts and autoregressive adjustment, controlling for sociodemographic and health covariates and the time between waves. Higher prior EURO-D scores predicted slightly lower subsequent cognitive performance, and higher prior cognition predicted fewer subsequent depressive symptoms; in both directions, cross-domain effects were small relative to within-domain stability. Domain-specific analyses indicated the most consistent prospective links for memory and orientation, with weaker evidence for numeracy. Grandparenthood (having grandchildren) was not associated with the cognitive composite but was associated with slightly lower depressive symptoms. As a secondary aim, interaction models tested whether grandparenthood moderated the lagged associations; interactions were small and sensitive to specification, suggesting modest heterogeneity (including a crossover pattern for cognition predicting depression) rather than a uniform “enrichment” effect. Overall, depressive symptoms and cognition show modest bidirectional prospective links, and grandparenthood status marks limited, non-causal differences in affect–cognition coupling within an observational design.

## 1. Introduction

Cognitive ageing is typically characterized by subtle declines in multiple cognitive abilities in many older adults. When measurable deficits emerge in one or more domains but daily functioning remains largely preserved, they are generally classified as mild cognitive impairment (MCI); when cognitive deficits become sufficiently severe to interfere with everyday functioning, they meet criteria for dementia (Dunne et al., 2021). Because late-life cognitive changes often unfold alongside affective symptoms, understanding their co-development has become central to research on functional ageing. Depressive symptoms are also common in later life—often precipitated by multimorbidity or severe life events such as bereavement—and remain substantially underdiagnosed and undertreated, leaving many older adults living with persistent symptoms. Importantly, late-life depressive symptoms and cognitive changes share clinical features, frequently co-occur, and exhibit bidirectional links across the life course: depression in earlier life elevates dementia risk; depression in later life may represent a prodromal manifestation of neurodegeneration, and cognitive dysfunction can also be attributable to depression (Fernández Fernández et al., 2024; Yin et al., 2024).

Depression is one of the most prevalent mental health conditions worldwide and represents a major contributor to disability across adulthood and later life (World Health Organization, 2025). Beyond its affective symptoms, depression is strongly associated with disturbances in several core cognitive abilities, including memory, attention, executive functioning, and information-processing speed, which may persist even after clinical remission (Ahern et al., 2025; Melendez et al., 2026; Rhee et al., 2024). At the same time, a growing body of longitudinal research demonstrates that depressive symptoms and cognitive functioning prospectively influence each other over time, forming a dynamic, bidirectional relationship with implications for functional ageing and risk of later impairment (Yin et al., 2024). These reciprocal associations imply that affective state and cognitive performance can act as both outcomes and antecedents of one another in late life, underscoring the importance of modeling temporal ordering and potential feedback loops in observational analyses.

Social and cognitively stimulating engagement in later life has consistently been associated with better cognitive functioning and a lower risk of cognitive decline, suggesting that social roles may constitute important contexts for maintaining cognitive health (Melendez et al., 2026). From a cognitive enrichment/cognitive reserve perspective, grandparenthood status may index exposure to socially embedded opportunities that can be cognitively stimulating, whereas evidence from grandparenting research suggests that well-being varies across role circumstances, with more demanding arrangements often linked to worse outcomes. Accordingly, any associations involving grandparenthood status are likely to reflect a mix of enrichment opportunities and potential role-related strain that cannot be disentangled with a binary indicator (Danielsbacka et al., 2022; Hu et al., 2025; Stafford et al., 2024).

Against this background, grandparenthood has received increasing attention as a meaningful social role that may provide opportunities for cognitively and socially stimulating engagement, with potential consequences for older adults’ emotional and cognitive well-being. While research suggests that many grandparents derive a sense of purpose, social engagement, and emotional fulfillment from interactions with grandchildren, findings regarding its consequences for mental health remain mixed. Systematic reviews show that non-coresiding or moderately involved grandparents often display better well-being, whereas custodial or intensive caregivers may experience higher stress and poorer mental health outcomes (Danielsbacka et al., 2022; Hu et al., 2025). Similarly, evidence concerning cognitive functioning is heterogeneous: some studies report that grandparenting—particularly through cognitively stimulating shared activities—may enhance cognitive performance, whereas others caution that high-frequency caregiving may be detrimental (Rafael et al., 2021).

Beyond global “grandparent versus non-grandparent” contrasts, recent longitudinal panel evidence indicates that the transition to grandparenthood as a life-course event is associated with higher cognitive functioning, with stronger effects among women and only modest between-strata variability once additive social determinants are accounted for, situating grandparenthood within a cognitive enrichment framework (Alonso-Pérez et al., 2024). Complementarily, propensity-matched growth-curve analyses from the English Longitudinal Study of Ageing suggest that among caregiving grandparents, caregiving frequency per se does not predict cognitive level or decline; instead, the nature and diversity of shared activities—e.g., cognitively engaging leisure or homework support—relate to higher cognitive levels, shifting emphasis from dose to content and variety (Chereches et al., 2026). This shift from mere frequency to the qualitative nature of the role suggests that the impact of grandparenthood may be best understood through the lens of person–role fit. This framework implies that the cognitive and emotional outcomes of a social role depend on the congruence between an individual’s internal resources—such as their cognitive reserve, reflected in life-course factors like education and cognitively stimulating experiences, and affective stability—and the specific demands and rewards of that role.

Building on this, the clinical overlap and potential bidirectionality between depressive symptoms and cognitive decline provides a strong rationale to test whether emotional health conditions the cognitive correlates of social roles such as grandparenthood. Crucially, emotional health may shape how grandparenthood relates to cognitive functioning, given well-established pathways linking depressive symptoms to poorer cognition and evidence that cognition can, in turn, influence vulnerability to depression (Ahern et al., 2025; Yin et al., 2024). Specifically, managing persistent distress may deplete the executive energy required to engage in stimulating social roles (Kanfer & Ackerman, 1989), potentially neutralizing the cognitive benefits of grandparenthood. Conversely, grandparenthood may be accompanied by greater meaning and generative purpose and may increase exposure to socially embedded, potentially cognitively engaging interactions, which could be associated with higher cognitive functioning when affective symptoms are lower—consistent with enrichment accounts—whereas under higher symptom burden, any advantage may be attenuated because depressive symptoms are prospectively linked to poorer cognitive performance and may constrain sustained engagement with demanding activities (Ahern et al., 2025; Kanfer & Ackerman, 1989; Yin et al., 2024). Importantly, because grandparenthood is operationalized here as a binary status indicator (having grandchildren), we interpret these expectations as status based associations rather than direct evidence about caregiving intensity, role quality, or activity content. Taken together, these strands imply that any cognitive benefits of grandparenthood status may be conditional on affective context and may also vary across role circumstances documented in prior grandparenting research (Alonso-Pérez et al., 2024; Chereches et al., 2026; Danielsbacka et al., 2022; Hu et al., 2025), with suggestive sex-specific advantages in some cohorts (Alonso-Pérez et al., 2024). These questions are particularly relevant in ageing populations, where both depression and cognitive decline impose substantial public-health burdens (World Health Organization, 2025).

At the same time, a rigorous account of potential confounders and stratifiers is essential. First, age is a primary determinant of both cognitive change and late-life mood, and the emotional correlates of grandparenting can vary by age band and contextual stressors (e.g., pandemic conditions), underscoring the need to adjust for and stratify by age where appropriate (Bünning & Huxhold, 2024; World Health Organization, 2025; Yin et al., 2024). Second, education is commonly used as a proxy indicator of cognitive reserve, reflecting life-course exposures associated with the development of reserve, and is associated with slower cognitive decline across multiple cohorts; moreover, lower education can amplify the deleterious association of depressive symptoms with cognition, suggesting that education is both a confounder and a plausible effect modifier (Luo et al., 2025; Cano-López et al., 2021; Yin et al., 2024). Collectively, these considerations motivate a modeling strategy that prioritizes time-ordered associations while adjusting for major sociodemographic and health-related correlates that may shape both depressive symptoms and cognition.

Third, income captures socioeconomic position that is tightly linked to depression and late-life cognitive disparities; failure to account for income risks conflating role-related differences with socioeconomic selection (Yin et al., 2024). Fourth, self-rated health (SRH)—a robust integrative marker—relates to subsequent cognition via depressive symptoms, indicating that SRH acts as a meaningful covariate (and potential pathway) in models that connect social roles to cognitive outcomes (Bae et al., 2024; Stafford et al., 2024). Finally, given evidence for sex-linked patterns in grandparenthood–cognition associations, sex should be modeled explicitly and considered in interaction tests (Alonso-Pérez et al., 2024; Yin et al., 2024). Collectively, these considerations motivate an analytic framework that controls for age, sex, education, income, and SRH when estimating time-ordered affect–cognition associations and when probing whether grandparenthood interacts with affect or cognition.

These processes are also shaped by broader socio-institutional contexts. Countries differ in health-care systems, educational structures, and norms surrounding intergenerational roles, which may influence both cognitive aging and depressive symptoms, as well as the meaning and practice of grandparenthood. Consequently, individual trajectories are embedded within national contexts that may condition the associations examined in this study (Bartucz et al., 2022; Noriega et al., 2020). A cross-national panel such as SHARE is therefore well positioned to examine whether observed associations generalize across heterogeneous contexts, while recognizing that cross-country differences may reflect both compositional and institutional sources.

The present study addresses a focused empirical gap: whether grandparenthood status is associated with differences in the reciprocal, time-ordered association between depressive symptoms and cognitive functioning in later life. Using large-scale, longitudinal data from older adults, we assess whether depressive symptoms and cognition mutually predict one another over time, and whether the cognitive advantage associated with grandparenthood varies depending on emotional well-being.

Hypotheses. First, we expect depressive symptoms to prospectively predict lower subsequent cognitive functioning, net of covariates (Ahern et al., 2025; Yin et al., 2024). Second, we expect cognitive functioning to prospectively predict fewer subsequent depressive symptoms, consistent with reciprocal associations observed in prior longitudinal work (Yin et al., 2024). Third (secondary, exploratory), we examine whether grandparenthood moderates these associations: consistent with cognitive enrichment accounts, we anticipate that any cognitive advantage associated with grandparenthood will be most evident at lower levels of depressive symptoms (i.e., depressive symptoms may attenuate the grandparenthood–cognition association), while emphasizing that—given the coarse measure—the direction and magnitude of moderation are evaluated cautiously and interpreted as status based differences rather than mechanistic effects (Alonso-Pérez et al., 2024; Chereches et al., 2026). By distinguishing the primary bidirectional question from the secondary moderation question and by aligning expectations with what the measures can support, the study aims to clarify how a common later life role intersects with two core psychological dimensions of ageing in a large observational panel. We treat moderation tests as exploratory and interpret them as status-based heterogeneity, given the binary operationalization of grandparenthood.

## 2. Materials and Methods

### 2.1. Study Design and Data Source

This study used a longitudinal observational design based on the Survey of Health, Ageing and Retirement in Europe (SHARE). SHARE is a multi-country panel of adults aged 50+ that administers consistent, repeated measures of health, socioeconomic conditions, and cognitive functioning, enabling within-person analyses across nine survey waves and cross-national comparisons from 2004 to 2022. No new primary data collection was undertaken. The present study used data from waves 2 and 4–9 collected between 2006/2007 and 2021/2022.

### 2.2. Participants

The analytic cohort comprised person-wave observations from participants aged 50 years or older with at least two consecutive waves, which is essential to derive first-order lags (t − 1) and to estimate temporal directionality. Starting from the initial SHARE Waves 1–9 file (N = 488,400 person-wave observations), applying the strict lagged requirement (t − 1 available and consecutive waves) yielded N = 231,461 observations (Lost = 256,939; exclusion of baseline waves with t–1 missing and single-wave participants). Inclusion required valid observations on cognitive functioning and depressive symptoms together with complete information on demographic, economic, and health covariates. SHARE specific negative codes for missingness (e.g., −1, −2, −3, −7, −15) were treated as missing according to the study’s coding guidelines. The final multilevel (mixed effects) model was therefore estimated on N = 53,015 observations, with 178,446 observations excluded due to listwise deletion (i.e., missingness in one or more model variables) (see Table A5).

### 2.3. Measures

Cognitive functioning was operationalized as a composite index (cognition index) that aggregates immediate and delayed word recall, numeracy, and orientation. Each component score was standardized within a wave (z-scores), and the index was computed as the mean of the standardized components so that higher values indicate better cognitive performance. Within-wave standardization supports comparability of relative standing within each wave, but it limits inference about absolute between-wave change. Although alternative dimensionality-reduction strategies (e.g., PCA) are common in SHARE applications, the present analysis adopted a transparent z-score averaging step to facilitate replication with harmonized files and consistent scaling across waves. This strategy aligns with prior SHARE applications focused on cognition and within-person change.

Depressive symptoms were assessed using the 12-item EURO-D scale (range 0–12), a validated instrument developed by a European consortium to enable cross-national comparisons of late-life depression. The scale assesses twelve symptom domains commonly observed in older adults—depression, pessimism, suicidality, guilt, sleep disturbance, loss of interest, irritability, appetite changes, fatigue, concentration difficulties, reduced enjoyment, and tearfulness (Prince et al., 1999). Higher scores indicate greater depressive symptomatology. Cross-national validation studies further support its measurement equivalence across diverse cultural contexts, confirming its suitability for comparative research in older adult populations (Maskileyson et al., 2021). EURO-D was modelled as a continuous measure in the mixed-effects framework. We inspected its distribution; floor effects are plausible in population samples and may compress variability, potentially attenuating prospective associations. Grandparenthood was coded at each wave as a binary status variable indicating whether respondents reported having at least one grandchild (0 = no grandchildren; 1 = at least one grandchild). This information was derived from the SHARE family roster, which records the number of grandchildren reported by each participant. Because SHARE follows individuals longitudinally across survey waves, this status could change over time for participants who became grandparents during the observation period. Covariates included age (years), sex (0 = male; 1 female), educational attainment (ISCED-97; 0 = no education; 1 = primary; 2 = lower secondary; 3 = upper secondary; 4 = post-secondary non-tertiary; 5 = first stage tertiary; 6 = doctoral level), net household income (continuous monetary measure), and self-rated health (1 = excellent to 5 = poor). These covariates were chosen for their theoretical relevance to cognitive and affective ageing.

To estimate temporal influence, first-order lagged variables were constructed after sorting records by unique individual identifier and wave. For each wave t, depression_lag and cognition_lag captured, respectively, the EURO-D and cognition values recorded at t − 1 for the same individual. This procedure constrained cross-lagged comparisons to strictly consecutive measurements, preserving the panel logic and reducing bias from variable inter-wave intervals. Such two-step lagging permits explicit baseline control within linear regression while retaining the interpretability expected in SHARE-based longitudinal analyses.

### 2.4. Statistical Analysis

The analytical strategy comprised three sets of linear mixed-effects models estimated in IBM SPSS Statistics (Version 30) using restricted maximum likelihood (REML). Given the hierarchical structure of the SHARE data, with repeated observations nested within individuals, a multilevel modeling approach with person-specific random intercepts was adopted to account for the non-independence of repeated observations.

Time elapsed between consecutive observations was additionally included as a covariate to account for variability in follow-up intervals across respondents. REML estimation was preferred because the primary objective was the estimation of variance components and fixed effects rather than formal likelihood-ratio comparisons between nested models. Linear mixed-effects models can accommodate unbalanced panels (different numbers of repeated observations) and do not require complete follow-up for all respondents. However, observations with missing values in model covariates/predictors were excluded from the corresponding analyses in SPSS, yielding complete-case estimation for the fixed-effect covariate set. We therefore highlight potential selection/attrition bias as a limitation.

In Model 1, cognitive functioning was regressed on lagged depressive symptoms while controlling for prior cognition and covariates (grandparenthood, age, sex, education, household income, self-rated health, and time between observations). To examine domain specificity, the model was replicated separately for individual cognitive outcomes (immediate recall, delayed recall, numeracy–percentage, numeracy–subtraction, and orientation).

In Model 2, depressive symptoms were regressed on prior cognitive functioning while controlling for prior depressive symptoms and the same covariates. Parallel models were estimated using lagged domain-specific cognitive measures. Continuous predictors involved in interaction terms were grand-mean centered prior to estimation to facilitate interpretation of lower-order effects and reduce multicollinearity.

Two additional moderation models examined whether grandparenthood moderated the longitudinal associations between depressive symptoms and cognitive functioning. In the first moderation model, cognitive functioning was regressed on depressive symptoms, grandparenthood, and their interaction (depressive symptoms × grandparenthood), adjusting for covariates. In the second moderation model, depressive symptoms were regressed on cognitive functioning, grandparenthood, and their interaction (cognition × grandparenthood), also adjusting for covariates. Simple slopes were derived algebraically from the fixed-effect estimates of the interaction models. Across models, fixed effects (Type III) are reported as unstandardized coefficients (B), standard errors, t-values, and *p*-values. Assumptions (linearity, homoscedasticity, normality, and multicollinearity) were checked using standard diagnostics. The inclusion of lagged predictors reflects a lagged multilevel regression framework with autoregressive adjustment, enabling the estimation of prospective associations conditional on prior levels of the outcome rather than a full structural equation modeling (SEM) cross-lagged panel design.

Given SHARE’s cross-national design, we conducted robustness checks by re-estimating Models 1 and 2 with an additional random intercept for respondents’ country of citizenship to account for national-level clustering. We also addressed heterogeneity in grandparent status by replacing the binary grandparenthood indicator with a three-category variable (grandparents status: 0 = stable non-grandparents; 1 = new grandparents; 2 = stable grandparents), modeled as a fixed effect with stable grandparents as the reference group.

Accordingly, estimates should be interpreted as time-ordered prospective associations with autoregressive adjustment, not as structural cross-lagged effects establishing causal direction.

## 3. Results

### 3.1. Model 1: Effects of Prior Depressive Symptoms on Later Cognitive Functioning

A linear mixed-effects model was estimated to examine the association between prior depressive symptoms and subsequent cognitive functioning, controlling for prior cognition and relevant covariates (grandparenthood status, age, sex, education, household income, time between observations and self-rated health).

Results indicated that prior cognitive functioning was by far the strongest longitudinal correlate of subsequent cognition, reflecting high temporal stability (see Table 1). In contrast, prior depressive symptoms were negatively associated with later cognitive functioning; however, this effect was small relative to the stability of cognition over time.

Among the covariates, female sex, educational attainment, and household income were positively associated with cognitive functioning. In contrast, age, longer time elapsed between measurements and poor self-rated health were negatively related to cognition. Grandparenthood status was not significantly associated with cognitive functioning.

Overall, these findings provide longitudinal evidence that depressive symptoms are longitudinally associated with subsequent cognitive functioning, above and beyond the strong temporal stability of cognition. No cognitive advantage was observed for grandparents in this model.

### 3.2. Model 2: Effects of Prior Cognitive Functioning on Later Depressive Symptoms

A linear mixed-effects model was estimated to examine whether prior cognitive functioning was associated with subsequent depressive symptoms, controlling for prior depressive symptoms and the same set of covariates.

Prior depressive symptoms emerged as the strongest longitudinal correlate of subsequent depressive symptoms, indicating substantial temporal stability in emotional well-being (see Table 2). Prior cognitive functioning was negatively associated with later depressive symptoms.

Among the covariates, female sex, and poor self-rated health were positively associated with depressive symptoms. In contrast, age, grandparenthood status, time between observations and educational attainment were negatively associated with depressive symptoms, indicating lower depressive symptom scores among grandparents in this specification. Household income was not significant predictor.

Taken together, these findings support a bidirectional—yet asymmetrical—longitudinal association between cognitive functioning and depressive symptoms. While both domains are prospectively associated with each other over time, within-domain stability is substantially stronger than cross-domain effects. Notably, grandparenthood was associated with lower depressive symptoms but not with cognitive functioning, suggesting domain-specific associations.

To address potential heterogeneity across cognitive domains, the main models were re-estimated separately for each cognitive component (see Appendix A Table A1 and Table A2). Overall, the results revealed broadly consistent—yet clearly domain-specific—patterns in the bidirectional associations between depressive symptoms and cognitive functioning.

When predicting cognitive outcomes (Table A1), prior depressive symptoms were negatively and significantly associated with memory and orientation domains (immediate recall, delayed recall, and orientation), whereas the evidence was weaker for numeracy outcomes (percentage and subtraction), where associations were small and not statistically significant (or only marginal). Across all domains, prior cognitive performance remained the strongest predictor, indicating substantial within-domain temporal stability.

When reversing the direction of the association (Table A2), prior cognitive functioning showed a robust prospective association with subsequent depressive symptoms for most cognitive domains, with immediate recall, delayed recall, numeracy–subtraction, and orientation being associated with later depressive symptoms, while numeracy–percentage showed no evidence of a prospective effect. The overall pattern indicates that cognitive functioning is prospectively associated with depressive symptoms, even if cross-domain effects remain modest relative to within-domain stability.

Taken together, these findings support the robustness of the main results while highlighting meaningful heterogeneity across cognitive domains. Although the overall pattern is consistent with a bidirectional relationship, the strength and statistical significance of these associations vary depending on the specific cognitive domain considered. Importantly, effect sizes were small across all models, reinforcing the conclusion that cross-domain influences are modest relative to the pronounced within-domain stability observed over time.

Sensitivity analyses yielded substantively unchanged conclusions: the lagged prospective association between prior depressive symptoms and later cognition remained statistically detectable but very small, prior cognition remained the dominant predictor, and grandparenthood showed no robust association with the cognitive composite once covariates were controlled.

Robustness checks were then conducted to test whether this grandparenthood pattern was sensitive to alternative specifications (Appendix B Table A4). When including a country-of-citizenship random intercept, the grandparenthood coefficient changed sign but remained very small in magnitude, suggesting that unobserved heterogeneity by citizenship may absorb part of the association. When operationalizing grandparenthood dynamically (stable non-grandparents; new grandparents; stable grandparents as reference), neither stable non-grandparents nor new grandparents differed significantly from stable grandparents, and the estimates for new grandparents were negative (albeit non-significant), which is compatible with the interpretation that becoming a grandparent is not associated with increased depressive symptoms and may even reflect a small—though imprecisely estimated—beneficial association. Overall, these robustness analyses suggest that any grandparenthood effect is likely modest and contingent on specification, but the data provide no consistent evidence that grandparenthood (including transitions into the role) increases depressive symptoms; instead, the pattern remains compatible with small favorable associations under the main model and with negligible differences under stricter/alternative definitions.

### 3.3. Model 3: Grandparenthood, Depressive Symptoms, and Cognitive Functioning (Moderation Analysis)

#### 3.3.1. Step 1: Depressive Symptoms as Moderator of Grandparenthood–Cognition Link

A linear mixed-effects model with person-specific random intercepts was estimated to examine whether depressive symptoms moderated the association between grandparenthood and cognitive functioning.

Prior depressive symptoms were not significantly associated with cognitive functioning (B = −0.0018, SE = 0.0013, t = −1.34, *p* = .179, 95% CI [−0.00435, 0.00075]), whereas grandparenthood was also not a significant predictor (B = −0.0052, SE = 0.0067, t = −0.78, *p* = .433, 95% CI [−0.01833, 0.00793]). Critically, the interaction between grandparenthood and depressive symptoms was statistically significant (B = 0.0028, SE = 0.0014, t = 2.05, *p* = .040, 95% CI [0.00006, 0.00554]), indicating that the association between depressive symptoms and cognitive functioning varied by grandparenthood status.

To aid interpretation beyond *p*-values, we report model-predicted cognition values by grandparenthood at representative values of lagged depressive symptoms and simple slopes by group derived from the fixed-effect covariance matrix (see Appendix D Table A6).

Simple slope analyses indicated that depressive symptoms were weakly and non-significantly associated with cognition among non-grandparents (slope = −0.0018). Among grandparents, however, the interaction term attenuated this negative association, yielding a slightly positive estimated slope (slope = −0.0018 + 0.0028 = 0.0010). Thus, the interaction pattern suggests that the depression–cognition association was slightly negative among non-grandparents and slightly positive among grandparents, which is more consistent with a modest attenuation or buffering pattern than with heightened vulnerability.

Given the extremely small magnitude of the interaction, this finding should be interpreted cautiously and primarily as evidence of heterogeneity in the depression–cognition association across grandparenthood groups rather than as evidence of a substantively large protective effect. Importantly, the time between observations was negatively associated with cognition (B = −0.0060, SE = 0.0004, t = −15.12, *p* < .001), underscoring the value of adjusting for measurement spacing in longitudinal models of cognitive change.

When re-estimating the same moderation model with a country-of-citizenship random intercept, the interaction remained statistically significant and slightly stronger (B = 0.0038, SE = 0.0013, t = 2.79, *p* = .005). In this specification, grandparenthood showed a small negative main effect (B = −0.0163, *p* = .008), while the interaction term remained positive, again suggesting that any grandparenthood-related differences are expressed primarily in how depressive symptoms relate to cognition rather than in a uniform cognitive advantage.

When grandparenthood was operationalized as transition/status categories (stable non-grandparents; new grandparents; stable grandparents as reference) and interacted with depressive symptoms, the overall interaction was not statistically significant (*p* = .432), and neither category-specific interaction term reached significance. This suggests that the moderation effect, while detectable in the main dichotomous specification, may be sensitive to how grandparenthood is defined (status vs. transition) and therefore should be framed as modest and not uniformly robust across operationalizations. Taken together, these findings indicate that grandparenthood does not confer a uniform cognitive advantage. Rather, the results are most consistent with small and specification-sensitive heterogeneity in the depression–cognition association, with some evidence suggesting that the negative association between depressive symptoms and cognition may be attenuated among grandparents.

#### 3.3.2. Step 2: Cognitive Functioning as Moderator of Grandparenthood–Depression Link

A second linear mixed-effects model with person-specific random intercepts was estimated to examine whether cognitive functioning moderated the association between grandparenthood and depressive symptoms.

Cognitive functioning was significantly associated with depressive symptoms (B = −0.645, SE = 0.115, t = −5.62, *p* < .001, 95% CI [−0.8704, −0.4196]), indicating fewer subsequent EURO-D scores at higher values of the cognitive predictor (as coded). The main effect of grandparenthood remained not statistically significant (B = −0.082, SE = 0.052, t = −1.57, *p* = .116, 95% CI [−0.1839, 0.0199]), suggesting no overall mean-level differences between grandparents and non-grandparents once covariates were controlled.

Importantly, the interaction between grandparenthood and cognitive functioning was statistically significant (B = 0.530, SE = 0.122, t = 4.40, *p* < .001, 95% CI [0.290, 0.770), indicating that the strength of the association between cognitive functioning and depressive symptoms varied by grandparenthood status.

We further summarize this interaction using predicted EURO-D values by grandparenthood at representative values of lagged cognition and simple slopes by group derived from the fixed-effect covariance matrix (see Appendix D Table A7).

Simple slope analyses indicated that higher cognitive functioning was associated with lower depressive symptoms among non-grandparents (slope = −0.645). Among grandparents, however, the interaction substantially attenuated this association, yielding a markedly weaker negative slope.

Accordingly, the interaction reflects substantial attenuation in the strength of the cognition–depression association across grandparenthood groups rather than a full reversal in direction. Whereas higher cognitive functioning was associated with lower depressive symptoms among non-grandparents, this negative association was markedly weaker among grandparents.

Taken together, these findings suggest that the association between cognitive functioning and depressive symptoms differs substantially by grandparenthood status. Although the overall longitudinal association observed in Model 2 indicated that better cognition was associated with fewer depressive symptoms, the moderation analyses reveal important heterogeneity across social roles. Therefore, the interaction is best interpreted as evidence that the cognition–depression relationship varies by grandparenthood status rather than as evidence of a uniformly protective effect of cognition among grandparents.

Notably, the time between observations was negatively associated with depressive symptoms (B = −0.049, SE = 0.0047, t = −10.44, *p* < .001, 95% CI [−0.0582, −0.0398]), underscoring the importance of adjusting for measurement spacing when modeling longitudinal change in EURO-D.

When re-estimating the model with a country-of-citizenship random intercept, the interaction remained significant (B = 0.820, SE = 0.121, t = 6.76, *p* < .001), and the main effect of grandparenthood remained non-significant (*p* = .175), indicating that the moderation effect was robust to this alternative specification. When operationalizing grandparenthood via transition/status categories, the interaction was again statistically significant overall (*p* < .001), with negative interaction contrasts for stable non-grandparents and new grandparents relative to stable grandparents, suggesting that the cognition–depression association differed across grandparenthood groups and was comparatively more positive among stable grandparents (the reference category).

Finally, the moderation effect in Step 2 remained clearly stronger (and more consistently robust across specifications) than the corresponding moderation in Step 1, suggesting greater robustness of the moderation effect in Step 2 relative to Step 1.

## 4. Discussion

The present study examined the bidirectional longitudinal associations between depressive symptoms and cognitive functioning in older adults and evaluated whether grandparenthood moderated these processes. Using time-ordered lagged modeling strategies within a multilevel framework, our findings extend existing evidence regarding the cognitive and emotional implications of late-life social roles and contribute to ongoing debates about the conditions under which grandparenthood may relate to healthy cognitive ageing.

Consistent with previous research, our results indicate that depressive symptoms and cognitive functioning are prospectively associated in both temporal directions. Higher depressive symptoms prospectively were prospectively associated with lower subsequent cognitive performance, even after adjustment for prior cognition and covariates. However, this effect was very small in magnitude relative to the strong temporal stability of cognition, indicating a limited longitudinal association at the within-person level. This pattern aligns with evidence that depression is associated with poorer performance in memory, executive functioning, and attentional control (Ahern et al., 2025; Fernández Fernández et al., 2024). Importantly, our domain-specific models refined this picture: prior depressive symptoms were reliably associated with later memory and orientation (immediate recall, delayed recall, and orientation), whereas evidence was weaker for numeracy (percentage and subtraction), where estimates were small and not statistically significant (or only marginal). This resonates with evidence that self-reported depressive symptoms are linked to performance decrements in tasks with higher attentional or mental-effort demands (Kessels et al., 2007). In our data, this pattern appeared most clearly for memory and orientation outcomes, while numeracy outcomes showed limited evidence of prospective associations. This is also consistent with broader clinical evidence that depressive symptoms are associated with poorer cognitive performance across multiple domains (Ahern et al., 2025; Rhee et al., 2024); however, our SHARE-based lagged models indicate domain specificity rather than uniform impairment, with the clearest prospective associations observed for memory and orientation. One plausible interpretation is consistent with resource-allocation accounts, which propose that performance on cognitively demanding tasks depends on the allocation of limited attentional resources (Kanfer & Ackerman, 1989). In the context of depressive symptoms, executive-control demands may be heightened by ruminative processing and reduced motivational/energetic capacity, which may be associated with poorer performance particularly on attentionally demanding cognitive tasks (Joormann & Gotlib, 2010). We emphasize, however, that this mechanism is speculative in the present observational design and cannot be directly tested with SHARE’s measures. This pattern is more consistent with affect-related disruptions in verbally mediated memory/orientation processes than with a broad decrement across all cognitive domains.

At a practical level, the magnitude of the depression–cognition association was extremely modest, especially compared with the autoregressive stability of cognition. Thus, although statistically detectable in a very large sample, changes in depressive symptoms were associated with only small subsequent differences in cognitive performance.

At the same time, the modest size of the effect suggests that depressive symptoms show a weak longitudinal association with cognitive change rather than a dominant driver. Although statistically significant, these cross-domain associations explain only a small proportion of variance, reinforcing the conclusion that cognition and depressive symptoms are only weakly coupled at the within-person level. Nevertheless, even small effects may accumulate over time or become relevant at the population level in ageing societies.

While previous landmark studies using the English Longitudinal Study of Ageing (ELSA) have established a bidirectional link between depressive symptoms and cognitive decline (Gale et al., 2012; Yin et al., 2019), our findings extend this evidence in several ways. First, by utilizing SHARE, we show that these reciprocal dynamics are observable across diverse European contexts. Second, consistent with newer large-scale longitudinal evidence, the bidirectionality appears asymmetrical, with strong within-domain stability (depression and cognition) and comparatively small cross-domain paths. (Yin et al., 2024). Third, rather than identifying a clear cognitive advantage associated with grandparenthood, our results suggest that its role is nuanced and emerges primarily through heterogeneity in associations—most clearly in interaction models—rather than through a uniform mean-level cognitive advantage.

Conversely, higher prior cognitive functioning was associated with lower subsequent depressive symptoms, which is compatible with the view that cognitive resources may buffer against late-life emotional distress In our main bidirectional model, this association was sizeable relative to the depression autoregressive path (e.g., cognition_lag B ≈ −1.41), indicating that lower prior cognitive functioning was consistently associated with higher subsequent EURO-D scores, net of prior EURO-D and covariates. These results align with theoretical models proposing dynamic feedback loops between cognitive and affective systems (Cacioppo & Hawkley, 2009), but the pattern also underscores that temporal ordering alone does not establish causality. Together, these results suggest that depressive symptoms and cognitive functioning are interrelated but largely stable domains that show relatively weak prospective associations over time.

A major contribution of this study is the analysis of whether grandparenthood moderates the association between depressive symptoms and cognitive functioning. In contrast to some prior literature, grandparenthood was not directly associated with cognitive functioning, indicating no overall cognitive advantage for grandparents in this sample. This null finding was consistent across the global cognition composite and across domain-specific models, where the grandparenthood main effect was generally small and non-significant once covariates and prior cognition were controlled. Accordingly, our results do not support a broad “cognitive enrichment” interpretation based solely on grandparent status (Alonso-Pérez et al., 2024), but they remain compatible with the idea that any cognitive advantages of grandparenthood may depend on the content and intensity of engagement—features not captured by SHARE’s binary grandparenthood indicator.

Moderation Step 1 suggested attenuation rather than heightened vulnerability. Specifically, the interaction term was positive and statistically significant but extremely small. Simple slopes indicated that depressive symptoms were weakly negative among non-grandparents, whereas the association was less adverse (and slightly positive) among grandparents once the interaction was added. Thus, the pattern is more consistent with a modest “buffering” or heterogeneity effect than with amplification of harm. Importantly, this interaction was sensitive to how grandparenthood was operationalized (status vs. transition categories) and should be interpreted cautiously as specification-dependent rather than as a robust protective mechanism.

Moderation Step 2 yielded a clearer and more robust interaction than Step 1. In this model, higher cognition predicted fewer depressive symptoms among non-grandparents, whereas among grandparents this negative association was substantially attenuated. Accordingly, the findings suggest that the cognition–depression association differs by grandparenthood status under this specification, rather than indicating a uniformly protective effect of cognitive functioning across groups. Potential explanations may include residual confounding, differences in social role expectations, or selection and measurement processes that vary across grandparenthood groups. Given the observational design and the relatively coarse operationalization of grandparenthood, these moderation findings should be interpreted as evidence of heterogeneity in prospective associations rather than as evidence for specific psychosocial mechanisms. Taken together, the moderation analyses indicate that grandparenthood does not operate as a simple protective factor but marks contextual heterogeneity in how cognition and depressive symptoms relate over time. Notably, the interaction in Step 2 was stronger and more consistently robust (including with country-of-citizenship random intercepts and alternative grandparent status coding) than the interaction in Step 1, supporting an asymmetry in moderation across the two temporal directions. These patterns are compatible with person–role fit accounts, in the sense that grandparenthood may mark contextual heterogeneity in resources and demands; however, the present operationalization cannot test role quality, caregiving intensity, or specific mechanisms. Future work should test whether the observed heterogeneity is concentrated among specific grandparenting configurations (e.g., custodial care, high-frequency care, or emotionally strained intergenerational ties), which are theoretically more likely to shape affect–cognition dynamics. This interpretation is consistent with research emphasizing the importance of activity content and engagement quality in shaping cognitive outcomes among grandparents (Chereches et al., 2026).

The observed associations remained robust after adjustment for age, sex, education, income, and self-rated health. Education was positively associated with cognition and showed small inverse associations with depressive symptoms in some models, consistent with its role as a proxy for cognitive reserve (Luo et al., 2025; Cano-López et al., 2021). In contrast to expectations, income was not a consistent predictor of depressive symptoms, suggesting that socioeconomic influences may operate differently across cognitive and emotional domains. Poor self-rated health emerged as a strong predictor of depressive symptoms, consistent with its role as an integrative indicator of vulnerability (Bae et al., 2024; Stafford et al., 2024).

Our results should be interpreted against the wider context in which late-life depressive symptoms and cognitive changes co-occur with multimorbidity, frailty, functional limitations, sleep disturbance, and neurobiological vulnerability. Integrative geriatric approaches such as comprehensive geriatric assessment illustrate how cognition and everyday functioning are embedded in broader physiological and nutritional profiles (Husejko et al., 2025), and contemporary reviews emphasize that depression and cognitive impairment overlap across the dementia continuum via vascular, inflammatory, and fronto-subcortical pathways (Jang et al., 2026). Sleep disturbance is also a key modifiable factor that relates to both mood and cognitive outcomes in older adults, and geriatric sleep reviews underscore downstream effects of sleep disruption on cognition and psychiatric symptoms (Tobias & Pisani, 2025). These perspectives reinforce that even small longitudinal associations—such as those observed here—may matter when embedded in accumulative risk profiles and interacting geriatric syndromes. Several limitations warrant consideration. First, although SHARE measures are well validated, they may not capture the full clinical complexity of depression or the multidimensional nature of cognition. Second, grandparenthood was operationalized as a binary status, without accounting for caregiving intensity, activity type, or relational quality (Danielsbacka et al., 2022). Third, the analytic design required strictly consecutive observations (to build t − 1 lags) and relied on complete-case estimation for the multivariable models; both choices may introduce selection/attrition bias if respondents with poorer health, higher depressive symptoms, or lower cognition are more likely to drop out or have missing covariate data. Fourth, given the large number of domain-specific and interaction tests, some statistically significant findings—especially very small interactions—may reflect multiplicity; accordingly, we emphasize pattern-level consistency and robustness checks rather than isolated *p*-values. Finally, although we included time between observations and explored country-level clustering, residual confounding (e.g., functional status, social support, caregiving burden, partner status, and health shocks) remains possible, constraining causal interpretation.

In summary, SHARE data provide evidence for small, asymmetrical bidirectional prospective associations between depressive symptoms and cognitive functioning in older adults, alongside modest and specification-sensitive moderation by grandparenthood. Grandparent status was not associated with a uniform cognitive advantage, but it was linked to slightly lower depressive symptoms in the main model and to heterogeneity in the cognition–depression coupling in moderation analyses. Future studies should move beyond binary grandparenthood toward measures of caregiving intensity, relationship quality, and role strain and should integrate geriatric factors such as sleep, frailty, and functional status to clarify when—and for whom—intergenerational roles may shape late-life affect–cognition dynamics.

## 5. Conclusions

This study provides evidence of a bidirectional, prospective—yet modest—longitudinal association between depressive symptoms and cognitive functioning in older adults. Across waves, within-domain stability was substantially stronger than cross-domain paths, indicating that changes in cognition and depressive symptoms are only weakly coupled over time once prior levels of the outcome are controlled. Consistent with the broader literature linking depression to cognitive performance, our results also highlight domain specificity, with the clearest prospective associations observed for memory and orientation, while numeracy showed weaker or null evidence of change.

Regarding grandparenthood, our findings do not support a uniform “cognitive enrichment” advantage associated with having grandchildren. Instead, grandparenthood appears primarily associated with contextual heterogeneity in how cognition and depressive symptoms relate over time. In the moderation analyses, the depression–cognition association was attenuated among grandparents in the main specification (a very small interaction), whereas the cognition–depression association displayed a stronger and more robust interaction, including a cross-over pattern that underscores qualitative differences between grandparents and non-grandparents in the cognition–depression coupling. Importantly, these moderation effects should be interpreted as heterogeneity in prospective associations rather than as evidence of specific psychosocial mechanisms, given the observational design and the coarse, binary measurement of grandparenthood. From a theoretical perspective, the results are most consistent with a conditional view of social roles: grandparenthood may be associated with contextual differences in how cognitive and emotional processes co-evolve rather than operating as an inherently protective (or harmful) exposure. Clinically and for public health, the findings reinforce that the cognitive–affective linkage in later life is detectable but small, and that interventions should be cautious about assuming that social engagement automatically translates into cognitive benefit. Supporting emotional well-being remains relevant, not because our models establish causality, but because mood symptoms and cognitive functioning are meaningfully interrelated over time in ageing populations. Finally, these conclusions should be considered alongside key limitations and priorities for future research. In particular, cross-national interpretation requires attention to measurement comparability and reporting differences in EURO-D across societies, and country-level moderators (e.g., welfare regimes, family systems, norms of symptom reporting) should be explicitly modeled when feasible. Future studies would benefit from moving beyond grandparenthood status to include caregiving intensity, contact frequency, co-residence, and relationship quality and from employing designs and methods that further reduce bias from attrition, missingness, and residual confounding.

## Figures and Tables

**Table 1:** Mixed-Effect model Predicting Cognitive Performance ^1^.

Predictor	B	SE	IC 95%	t	*p*
Prior cognition (lag)	0.243	0.004	[0.23516, 0.25084]	55.372	<.001
Prior depressive symptoms (lag)	−0.004	0.0005	[−0.00498, −0.00302]	−8.864	<.001
Grandparenthood	0.004	0.005	[−0.00580, 0.01380]	0.733	.463
Age (years)	−0.011	0.0002	[−0.011392, −0.010608]	−50.536	<.001
Female sex	0.031	0.004	[0.02316, 0.03884]	7.921	<.001
Education (ISCED)	0.013	0.001	[0.01104, 0.01496]	13.945	<.001
Household income	6.33 × 10^−8^	2.82 × 10^−8^	[8.03 × 10^−9^, 1.19 × 10^−7^]	2.243	.025
Poor self-rated health	−0.035	0.002	[−0.03892, −0.03108]	−22.474	<.001
Time between observations	−0.006	0.0004	[−0.006743, −0.005195]	−15.113	<.001

^1^ Estimates correspond to fixed effects from a linear mixed-effects Model 1.

**Table 2:** Mixed-Effect model Predicting Depressive Symptoms ^1^.

Predictor	B	SE	IC 95%	t	*p*
Prior depressive symptoms (lag)	0.307	0.005	[0.29720, 0.31680]	61.51	<.001
Prior cognition (lag)	−1.410	0.046	[−1.50016, −1.31984]	−30.95	<.001
Grandparenthood	−0.093	0.047	[−0.18512, −0.00088]	−2.00	.046
Age (years)	−0.025	0.002	[−0.02892, −0.02108]	−13.08	<.001
Female sex	0.529	0.033	[0.46432, 0.59368]	15.86	<.001
Education (ISCED)	−0.015	0.008	[−0.03068, 0.00068]	−2.06	.040
Household income	−9.65 × 10^−8^	3.14 × 10^−7^	[−7.12 × 10^−7^, 5.19 × 10^−7^]	−0.31	.759
Poor self-rated health	0.371	0.016	[0.33964, 0.40236]	23.89	<.001
Time between observations	−0.049	0.005	[−0.05880, −0.03920]	−10.38	<.001

^1^ Model 2.

## Data Availability

All data are available to registered researchers through the SHARE Research Data Center (www.share-project.org), accessed on 15 February 2026. No proprietary or restricted-access sources were used.

## Abbreviations

The following abbreviations are used in this manuscript:

EURO-D	European Depression Scale
ISCED	International Standard Classification of Education
MCI	Mild Cognitive Impairment
PCA	Principal Component Analysis
SPSS	Statistical Package for the Social Sciences
SRH	Self-Rated Health

## Appendix A

**Table A1:** Mixed-effects models predicting each cognitive component from prior depressive symptoms and covariates.

Cognitive Domain	Predictor	B	SE	t	*p*
Immediate Recall	Prior depressive symptoms (lag)	−0.011593	0.000844	−13.735	<.001
	Prior immediate recall (lag)	0.485962	0.007722	62.929	<.001
	Grandparenthood	0.010178	0.008431	1.207	.227
	Age (years)	−0.019489	0.000358	−54.491	<.001
	Female sex	0.062755	0.006343	9.894	<.001
	Education (ISCED)	0.020667	0.001444	14.317	<.001
	Household income	1.692 × 10^−7^	5.177 × 10^−7^	3.270	.001
	Poor self-rated health	−0.066316	0.002684	−24.704	<.001
	Time between observations	−0.013035	0.000739	−17.632	<.001
Delayed Recall	Prior depressive symptoms (lag)	−0.008550	0.000863	−9.906	<.001
	Prior delayed recall (lag)	0.502583	0.007898	63.631	<.001
	Grandparenthood	0.003760	0.008658	0.434	.664
	Age (years)	−0.022022	0.000368	−59.879	<.001
	Female sex	0.086563	0.006534	13.248	<.001
	Education (ISCED)	0.025420	0.001488	17.088	<.001
	Household income	2.141 × 10^−7^	5.281 × 10^−7^	4.054	<.001
	Poor self-rated health	−0.074304	0.002748	−27.037	<.001
	Time between observations	−0.014643	0.000753	−19.457	<.001
Numeracy–Percentage	Prior depressive symptoms (lag)	−0.000515	0.000290	−1.780	.075
	Prior numeracy (%) lag	0.12258	0.002645	4.633	<.001
	Grandparenthood	−0.000353	0.002674	−0.132	.895
	Age (years)	−0.000424	0.000111	−3.801	<.001
	Female sex	−0.006967	0.001905	−3.658	<.001
	Education (ISCED)	−0.000555	0.000428	−1.295	.195
	Household income	−1.123 × 10^−8^	1.828 × 10^−8^	−0.614	.539
	Poor self-rated health	0.003260	0.000897	3.633	<.001
	Time between observations	−0.001285	0.000277	−4.642	<.001
Numeracy–Subtraction	Prior depressive symptoms (lag)	−0.000129	0.000453	−0.286	.775
	Prior subtraction (lag)	0.164549	0.004157	39.582	<.001
	Grandparenthood	0.006846	0.005109	1.340	.180
	Age (years)	−0.007546	0.000228	−33.136	<.001
	Female sex	−0.013923	0.004252	−3.274	.001
	Education (ISCED)	0.013834	0.000973	14.220	<.001
	Household income	7.749 × 10^−8^	2.548 × 10^−8^	3.041	.002
	Poor self-rated health	−0.024833	0.001478	−16.805	<.001
	Time between observations	−0.002678	0.000354	−7.575	<.001
Orientation	Prior depressive symptoms (lag)	−0.006229	0.000594	−10.492	<.001
	Prior orientation (lag)	0.324682	0.005425	59.849	<.001
	Grandparenthood	−0.002316	0.005483	−0.422	.673
	Age (years)	−0.003242	0.000229	−14.188	<.001
	Female sex	0.008257	0.003905	2.115	.034
	Education (ISCED)	−0.000706	0.000878	−0.803	.422
	Household income	1.195 × 10^−7^	3.750 × 10^−7^	3.188	.001
	Poor self-rated health	−0.009474	0.001840	−5.149	<.001
	Time between observations	−0.000098	0.000568	−0.173	.863

**Table A2:** Mixed-effects models predicting depressive symptoms from prior cognitive components and covariates.

Cognitive Domain	Predictor	B	SE	t	*p*
Immediate Recall	Prior depressive symptoms (lag)	0.283707	0.005124	55.363	<.001
	Prior immediate recall (lag)	−1.005298	0.030169	−33.323	<.001
	Grandparenthood	−0.08301	0.046712	−1.777	0.076
	Age (years)	−0.014	0.001992	−7.029	<.001
	Female sex	0.521138	0.033583	15.518	<.001
	Education (ISCED)	−0.02405	0.007592	−3.168	0.002
	Household income	−7.48 × 10^−8^	3.14 × 10^−7^	−0.238	0.812
	Poor self-rated health	0.368988	0.015477	23.841	<.001
	Time between observations	−0.01337	0.004526	−2.955	0.003
Delayed Recall	Prior depressive symptoms (lag)	0.295924	0.005053	58.563	<.001
	Prior delayed recall (lag)	−0.821254	0.027932	−29.402	<.001
	Grandparenthood	−0.091073	0.046917	−1.941	0.052
	Age (years)	−0.01452	0.002009	−7.228	<.001
	Female sex	0.506032	0.033785	14.978	<.001
	Education (ISCED)	−0.02345	0.007649	−3.066	0.002
	Household income	−1.23 × 10^−7^	3.15 × 10^−7^	−0.39	0.696
	Poor self-rated health	0.361098	0.015557	23.211	<.001
	Time between observations	−0.01374	0.004533	−3.03	0.002
Numeracy–Percentage	Prior depressive symptoms (lag)	0.342089	0.004815	71.045	<.001
	Prior numeracy–percentage (lag)	−0.01522	0.081136	−0.188	0.851
	Grandparenthood	0.101555	0.047412	2.142	0.032
	Age (years)	−0.0276	0.001981	−13.934	<.001
	Female sex	0.544642	0.034162	15.943	<.001
	Education (ISCED)	0.006835	0.00767	0.891	0.373
	Household income	3.01 × 10^−7^	3.17 × 10^−7^	0.95	0.342
	Poor self-rated health	0.26727	0.015369	17.39	<.001
	Time between observations	−0.01	0.004569	−2.188	0.029
Numeracy–Subtraction	Prior depressive symptoms (lag)	0.313732	0.00499	62.867	<.001
	Prior numeracy–subtraction	−1.174048	0.052352	−22.426	<.001
	Grandparenthood	0.094177	0.047051	2.002	0.045
	Age (years)	−0.02262	0.001977	−11.44	<.001
	Female sex	0.598336	0.033946	17.626	<.001
	Education (ISCED)	−0.01117	0.007641	−1.462	0.144
	Household income	6.17 × 10^−8^	3.16 × 10^−7^	0.195	0.845
	Poor self-rated health	0.325069	0.01549	20.986	<.001
	Time between observations	−0.01219	0.004548	−2.679	0.007
Orientation	Prior depressive symptoms (lag)	0.344504	0.004807	71.669	<.001
	Prior orientation (lag)	−0.171813	0.014867	−11.557	<.001
	Grandparenthood	0.103572	0.047137	2.197	0.028
	Age (years)	−0.03213	0.002009	−15.997	<.001
	Female sex	0.538219	0.033888	15.882	<.001
	Education (ISCED)	0.007526	0.007604	0.99	0.322
	Household income	2.5 × 10^−7^	3.17 × 10^−7^	0.79	0.430
	Poor self-rated health	0.28452	0.015388	18.49	<.001
	Time between observations	−0.03142	0.004931	−6.371	<.001

## Appendix B

**Table A3:** Robustness checks for Model 1.

**Panel A. Model 1 with Country-of-Citizenship Random Intercept**				
**Predictor**	**B**	**SE**	**t**	***p***
Prior depressive symptoms (lag)	−0.006628	0.000477	−13.899	<.001
Prior cognition (lag)	0.410726	0.004636	88.591	<.001
Grandparenthood	−0.004549	0.004432	−1.027	.305
Age (years)	−0.009013	0.000185	−48.666	<.001
Female sex	0.017876	0.003108	5.751	<.001
Education (ISCED)	0.006640	0.000720	9.217	<.001
Household income	5.338 × 10^−8^	3.050 × 10^−8^	1.750	.080
Poor self-rated health	−0.035293	0.001512	−23.346	<.001
Time between observations	−0.009806	0.000470	−20.851	<.001
**Panel B. Model 1 with Grandparents Status (0 = Stable Non-Grandparents; 1 = New Grandparents; 2 = Stable Grandparents)**				
**Predictor**	**B**	**SE**	**t**	***p***
Prior depressive symptoms (lag)	−0.004550	0.000480	−9.474	<.001
Prior cognition (lag)	0.247555	0.004397	56.297	<.001
Stable non-grandparents	−0.003607	0.005442	−0.663	.507
New grandparents	−0.006382	0.007899	−0.808	.419
Age (years)	−0.011093	0.000221	−50.126	<.001
Female sex	0.031088	0.003961	7.848	<.001
Education (ISCED)	0.012600	0.000901	13.978	<.001
Household income	6.226 × 10^−8^	2.821 × 10^−8^	2.207	.027
Poor self-rated health	−0.033985	0.001548	−21.959	<.001
Time between observations	−0.006176	0.000397	−15.537	<.001

**Table A4:** Robustness checks for Model 2.

**Panel A. Model 2 with Country-of-Citizenship Random Intercept**				
**Predictor**	**B**	**SE**	**t**	***p***
Prior depressive symptoms (lag)	0.314063	0.005000	62.813	<.001
Prior cognition (lag)	−1.500089	0.048226	−31.106	<.001
Grandparenthood	−0.093020	0.046465	−2.002	.045
Age (years)	−0.024317	0.001940	−12.535	<.001
Female sex	0.519753	0.032624	15.932	<.001
Education (ISCED)	−0.012058	0.007544	−1.598	.110
Household income	−3.84 × 10^−7^	3.20 × 10^−7^	−1.202	.229
Poor self-rated health	0.364645	0.015818	23.052	<.001
Time between observations	−0.051343	0.004914	−10.448	<.001
**Panel B. Model 2 with Grandparents Status (0 = Stable Non-Grandparents; 1 = New Grandparents; 2 = Stable Grandparents)**				
**Predictor**	**B**	**SE**	**t**	***p***
Prior depressive symptoms (lag)	0.308101	0.005006	61.548	<.001
Prior cognition (lag)	−1.419049	0.045743	−31.022	<.001
Stable non-grandparents	−0.079697	0.048723	−1.636	.102
New grandparents	−0.152211	0.085904	−1.772	.076
Age (years)	−0.026048	0.001970	−13.219	<.001
Female sex	0.524905	0.033478	15.679	<.001
Education (ISCED)	−0.015677	0.007512	−2.087	.037
Household income	−1.05 × 10^−7^	3.14 × 10^−7^	−0.333	.739
Poor self-rated health	0.379142	0.015583	24.331	<.001
Time between observations	−0.050042	0.004731	−10.577	<.001

## Appendix C

**Table A5:** Sample selection and retained person-wave observations across analytic steps.

Step	Observations (N)	Lost (Step)	% Retained (Step)	% Retained (Cumulative)	Reason for Exclusion
Initial Sample	488,400	—	100.0%	100.0%	Total observations in SHARE Waves 1–9
Lagged Modeling (eligible for t − 1)	231,461	256,939	47.4%	47.4%	Exclusion of baseline waves (t − 1 missing) and single-wave participants
Final Model (complete-case multivariable)	53,015	178,446	22.9%	10.9%	Listwise deletion in the multilevel model (missingness in ≥1 model variable)

## Appendix D

**Table A6:** Model-predicted cognition and simple slopes by grandparenthood.

Predicted Values (EMMEANS) at Representative Values of Lagged Depression (Centered)		
Lagged Depression (Centered)	Non-Grandparents (0): Predicted Cognition	Grandparents (1): Predicted Cognition
−1.70	0.155	0.151
2.30	0.148	0.155
6.30	0.141	0.159
Simple slopes		
Group	Slope B	IC95%
Grandparents (1)	0.0010	[−0.00274, 0.00474]
Non-grandparents (0)	−0.0018	[−0.00435, 0.00075]

**Table A7:** Model-predicted depression and simple slopes by grandparenthood.

Predicted Values (EMMEANS) at Representative Values of Lagged Cognition (Centered)		
Lagged Cognition (Centered)	Non-Grandparents (0): Predicted Depression	Grandparents (1): Predicted Depression
−0.72	2.020	1.550
0.00	1.555	1.473
0.72	1.090	1.390
Simple slopes		
Group	Slope B	IC95%
Grandparents (1)	−0.110	[−0.340, 0.120]
Non-grandparents (0)	−0.645	[−0.870, −0.419]
